# Measurable residual disease, FLT3‐ITD mutation, and disease status have independent prognostic influence on outcome of allogeneic stem cell transplantation in NPM1‐mutated acute myeloid leukemia

**DOI:** 10.1002/cam4.4218

**Published:** 2022-01-20

**Authors:** Rama Al Hamed, Myriam Labopin, Etienne Daguindau, Riitta Niittyvuopio, Anne Huynh, Gerard Socié, Micha Srour, Jean Henri Bourhis, Nicolaus Kröger, Eleni Tholouli, Goda Choi, Xavier Poiré, Hans Martin, Marie‐Thérèse Rubio, Pavel Jindra, Didier Blaise, Dietrich Beelen, Hélène Labussière‐Wallet, Arnon Nagler, Ali Bazarbachi, Mohamad Mohty

**Affiliations:** ^1^ Department of Internal Medicine Jacobi Medical Center/Albert Einstein College of Medicine Bronx New York USA; ^2^ Department of Hematology EBMT Paris Office CEREST‐TC Saint Antoine Hospital Paris France; ^3^ Hôpital Jean Minjoz Service d`Hématologie Besançon France; ^4^ HUCH Comprehensive Cancer Center Stem Cell Transplantation Unit Helsinki Finland; ^5^ CHU ‐ Institut Universitaire du Cancer Toulouse Oncopole, Toulouse France; ^6^ Department of Hematology – BMT Hôpital St. Louis Paris France; ^7^ CHU de Lille Université de Lille Lille France; ^8^ Department of Hematology Gustave Roussy Cancer Campus BMT Service Villejuif France; ^9^ University Hospital Eppendorf Bone Marrow Transplantation Centre Hamburg Germany; ^10^ Clinica Haematology Department Manchester Royal Infirmary Manchester UK; ^11^ Department of Hematology University Medical Center Groningen (UMCG) University of Groningen Groningen The Netherlands; ^12^ Department of Haematology Cliniques Universitaires St. Luc Brussels Belgium; ^13^ Goethe‐Universitaet Medizinische Klinik II Hämatologie, Medizinische Onkologie Frankfurt Germany; ^14^ CHRU BRABOIS Service Hématologie Vandoeuvre‐lès‐Nancy France; ^15^ Department of Hematology/Oncology Charles University Hospital Pilsen Czech Republic; ^16^ Programme de Transplantation & Therapie Cellulaire Centre de Recherche en Cancérologie de Marseille Marseille France; ^17^ Department of Bone Marrow Transplantation University Hospital Essen Germany; ^18^ Centre Hospitalier Lyon Sud Service Hématologie Lyon France; ^19^ Hematology Division Chaim Sheba Medical Center Tel Hashomer Israel; ^20^ Department of Internal Medicine American University of Beirut Beirut Lebanon

**Keywords:** acute myeloid leukemia, FLT3‐ITD, minimal residual disease, NPM‐1 mutation

## Abstract

*Nucleophosmin*‐*1* (*NPM1*) mutations in acute myeloid leukemia (AML) confer a survival advantage in the absence of *FLT3*‐internal tandem duplication (*FLT3*‐ITD). Here, we investigated the main predictors of outcome after allogeneic hematopoietic stem cell transplantation (allo‐HCT). We identified 1572 adult (age ≥ 18 year) patients with *NPM1*‐mutated AML in first complete remission (CR1:78%) or second complete remission (CR2:22%) who were transplanted from matched sibling donors (30.8%) or unrelated donors (57.4%) between 2007 and 2019 at EBMT participating centers. Median follow‐up for survivors was 23.7 months. *FLT3*‐ITD was present in 69.3% of patients and 39.2% had detectable minimal/measurable residual disease (MRD) at transplant. In multivariate analysis, relapse incidence (RI) and leukemia‐free survival (LFS) were negatively affected by concomitant *FLT3*‐ITD mutation (HR 1.66 *p* = 0.0001, and HR 1.53, *p* < 0.0001, respectively), MRD positivity at transplant (HR 2.18, *p* < 10^−5^ and HR 1.71, *p* < 10^−5^, respectively), and transplant in CR2 (HR 1.36, *p* = 0.026, and HR 1.26, *p* = 0.033, respectively), but positively affected by Karnofsky score ≥90 (HR 0.74, *p* = 0.012, and HR 0.7, *p* = 0.0002, respectively). Overall survival (OS) was also negatively influenced by concomitant *FLT3*‐ITD (HR 1.6, *p* = 0.0001), MRD positivity at transplant (HR 1.61, *p* < 10^−5^), and older age (HR 1.22 per 10 years, *p* < 0.0001), but positively affected by matched sibling donor (unrelated donor: HR 1.35, *p* = 0.012; haploidentical donor: HR 1.45, *p* = 0.037) and Karnofsky score ≥90 (HR 0.73, *p* = 0.004). These results highlight the independent and significant role of *FLT3*‐ITD, MRD status, and disease status on posttransplant outcomes in patients with *NPM1*‐mutated AML allowing physicians to identify patients at risk of relapse who may benefit from posttransplant prophylactic interventions.

## Graphical abstract

Acute myeloid leukemia (AML) is a heterogenous disease with a highly variable prognosis and high overall mortality whereby the management is influenced by patient characteristics and biological, genetic, and molecular characteristics of the disease. The impact of minimal residual disease (MRD), NPM‐1 mutation status, FLT3‐ITD mutation status, and disease status at transplant has been individually investigated. This study examines the aggregate influence of those factors on outcome post‐transplant in NPM1‐mutated AML.

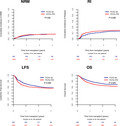

## 1 INTRODUCTION

Acute myeloid leukemia (AML) is a heterogenous disease with a highly variable prognosis and high overall mortality. The management of AML is guided by patient characteristics including age and performance status as well as biological, genetic, and molecular characteristics of the disease.^1^, ^2^ *Nucleophosmin*‐*1* (*NPM1*) is an essential gene encoding a protein that physiologically shuttles between the nucleus and cytoplasm to establish multiple protein–protein interactions involving critical cell functions such as formation and export of ribosomes, stabilization of the oncosuppressor p14^Arf^ protein, and regulation of centrosome duplication.^3^, ^4^, ^5^ *NPM1* mutations occur in approximately 30% of adult AML cases and in 50%–60% of normal karyotype AML, thus representing one of the most frequently encountered molecular abnormalities in AML.^5^, ^6^, ^7^ In normal karyotype AML, and in the absence of *FLT3*‐internal tandem duplication (*FLT3*‐ITD), *NPM1* mutation reduces the risk of relapse and confers a survival advantage. The entity is thus classified in the favorable risk group as per the European LeukemiaNet (ELN) 2017 classification.^2^, ^8^, ^9^ As such, allogeneic hematopoietic stem cell transplant (allo‐HCT) is deferred in these patients in first complete remission (CR1) unless in the setting of minimal/measurable residual disease (MRD) positivity or disease relapse.^10^ Conversely, when *NPM1* is present in the setting of *FLT3*‐ITD at high allelic burden, disease prognosis significantly decreases and patients are referred for allo‐HCT in CR1 irrespective of MRD status.^10^ Transplant indication in CR1 remains controversial in patients with *NPM1*‐mutated AML and low allelic burden of *FLT3*‐ITD.

Despite the continued progress in transplant techniques, approximately one third of AML patients relapse within 2 years of transplant which prompts the need for a better understanding of factors that influence outcome in this patient population. Current data suggest that the concomitant presence of *FLT3*‐ITD and *NPM1*, MRD positivity before or after transplant, and disease status at the time of transplant influence the risk of posttransplant relapse and thus affect outcome.^10^, ^11^, ^12^, ^13^ Nonetheless, the respective and independent contributions of these factors remain largely unknown. Using a large sample from the European Society for Blood and Marrow Transplantation (EBMT) registry, we investigated the predictive factors of posttransplant outcomes in patients with normal karyotype *NPM1*‐mutated AML with specific emphasis on the individual and aggregate roles of MRD status before transplant, *FLT3*‐ITD mutation status, and disease status at transplant.

## 2 MATERIALS AND METHODS

### 2.1 Study design and data collection

This was a retrospective, registry‐based, multicenter analysis.^14^ Data were provided and approved by the Acute Leukemia Working Party (ALWP) of the EBMT.^14^ The EBMT is a voluntary collaborative working group that includes more than 600 transplant centers that report all consecutive stem cell transplantations and follow‐up once a year, with regular audits to determine and maintain the accuracy of the data. Since 1 January 2003 and as per the Declaration of Helsinki of 1975, all transplant centers have been required to obtain written informed consent prior to data registration with the EBMT.

Eligibility criteria for this analysis included adult patients (age ≥18 years) diagnosed with normal karyotype, *NPM1*‐mutated AML, who received a first allo‐HCT between January 2007 and July 2019 and who had *FLT3*‐ITD mutation status and MRD status at the time of transplant, available in the EBMT registry. Variables collected included recipient and donor age, gender, and cytomegalovirus (CMV) serostatus, *FLT3*‐ITD mutation status, Karnofsky performance status (KPS) score, disease status, and MRD status at the time of transplant. Transplant‐related factors included year of transplant, conditioning regimen, donor type and degree of mismatch, source of stem cells, graft‐versus‐host disease (GVHD) prophylaxis, in vivo T‐cell depletion, and use of posttransplant cyclophosphamide.

### 2.2 Definitions

Myeloablative conditioning (MAC) was defined as a regimen containing either total body irradiation conditioning (TBI) with a dose equal or greater than 8 Gy, a total dose of oral busulfan (Bu) greater than 8 mg/kg, or a total dose of intravenous Bu greater than 6.4 mg/kg. All other regimens were defined as reduced intensity conditioning (RIC).^15^ Diagnosis and grading of acute^16^ and chronic GVHD^17^ were performed by transplant centers using standard criteria. High‐resolution HLA allele typing at loci A, B, C, DRB1, and DQ was retrieved from the EBMT registry for both the patient and the donor.

### 2.3 Endpoints

The primary endpoint of our study was assessing the predictive factors of leukemia‐free survival (LFS) post allo‐HCT. Secondary endpoints were overall survival (OS), relapse incidence (RI), non‐relapse mortality (NRM), acute and chronic GVHD, and GVHD‐free, relapse‐free survival (GRFS). LFS was defined as survival without disease relapse or progression whereby patients were censored at the time of last follow‐up. OS was defined as the time until death from any cause and NRM was defined as being alive until death without leukemia relapse. GRFS was defined as being alive with neither grades III–IV acute GVHD, extensive chronic GVHD nor relapse.^18^

### 2.4 Statistical analysis

Patient, disease, and transplant‐related characteristics were compared using χ^2^ statistics for categorical variables and the Mann–Whitney test for continuous variables. Probabilities of OS, LFS, and GRFS were calculated using the Kaplan–Meier method. Cumulative incidence was used to estimate the endpoints of NRM, RI, and acute and chronic GVHD to accommodate for competing risks. To study acute and chronic GVHD, we considered relapse and death to be competing events. A Cox proportional hazards model was used for multivariate regression. All factors associated with one outcome in univariate analysis were included in the Cox model. Results were expressed as a hazard ratio (HR) with a 95% confidence interval (CI). We also performed a univariate analysis separately in CR1 and CR2 patients. All tests were two sided. All analyses were performed using SPSS 24.0 (SPSS Inc, Chicago, IL, USA) and R version 3.6.2 (R Core Team. R: a language for statistical computing. 2014. R Foundation for Statistical Computing, Vienna, Austria).

## 3 RESULTS

### 3.1 Patient and transplantation characteristics

We identified a total of 1572 patients (53.9% female; median age 53.7 years [range 18.3–77.2]) who met the inclusion criteria––966 out of an original 2538 were excluded for lack of MRD status pre‐transplant. The majority (78%) of the patients were in CR1, with the remaining 22% in second complete remission (CR2). The KPS was ≥80 and ≥90 in 96.6% and 77.6% of the patients, respectively, at the time of transplant, and most (69.3%) of the patients harbored *FLT3*‐ITD mutation including 78.5% of patients transplanted in CR1 and 36.7% of patients transplanted in CR2 (Table 1). Table 1 also shows the comparison of transplant characteristics between CR1 and CR2 patients. Median follow‐up for survivors was 23.7 (interquartile range (IQR) 12–43.8) months and median year of transplant was 2016 (range: 2007–2019).

**TABLE 1:** Patient and transplant characteristics in CR1 and CR2 NPM1‐mutated AML.

		CR1 (*n* = 1226)	CR2 (*n* = 346)	Population (*n* = 1572)
Follow‐up (months)	Median (IQR)	24.1 (12–44.3)	21.7 (12–42.1)	23.7 (12–43.8)
Patient sex	Male	553 (45.1%)	170 (49.1%)	723 (46.1%)
Patient age at transplant (years)	Median (min–max) [IQR]	53.1 (18.3–76.4) [44.5–60.2]	55.5 (18.9–77.2) [46–63.4]	53.7 (18.3–77.2) [44.8–60.9]
CMV serostatus	Negative	411 (33.9%)	125 (36.4%)	536 (34.4%)
	Positive	803 (66.1%)	218 (63.6%)	1021 (65.6%)
	Missing	12	3	15
Time to transplant (months)	Median (min–max)[IQR]	4.9 (2–17.2)[3.9–6.1]	17.5 (3–126.7)[13.3–25.1]	
Year of transplant	Median (min–max)[IQR]	2015 (2010–2018)	2017 (2007–2019)	2016 (2007–2019) [2014–2018]
MRD at transplant	MRD negative	767 (62.6%)	189 (54.6%)	956 (60.8%)
	MRD positive	459 (37.4%)	157 (45.4%)	616 (39.2%)
*FLT3*‐ITD	*FLT3* wt	263 (21.5%)	219 (63.3%)	482 (30.7%)
	*FLT3‐ITD*	963 (78.5%)	127 (36.7%)	1090 (69.3%)
Karnofsky score	<80	35 (3%)	16 (4.9%)	51 (3.4%)
	>=80	1134 (97%)	308 (95.1%)	1442 (96.6%)
	Missing	57	22	79
Karnofsky score	<90	246 (21.1%)	88 (27.2%)	334 (22.4%)
	>=90	921 (78.9%)	236 (72.8%)	1157 (77.6%)
	Missing	59	22	81
Donor type	MSD	406 (33.1%)	78 (22.5%)	484 (30.8%)
	UD 10/10	407 (33.2%)	123 (35.5%)	530 (33.7%)
	UD 9/10	81 (6.6%)	39 (11.3%)	120 (7.6%)
	UD HLA missing	207 (16.9%)	46 (13.3%)	253 (16.1%)
	Haploidentical	125 (10.2%)	60 (17.3%)	185 (11.8%)
Donor sex	Male	809 (66.3%)	222 (64.5%)	1031 (65.9%)
	Female	411 (33.7%)	122 (35.5%)	533 (34.1%)
	Missing	6	2	8
Female to male allograft	No female to male	1071 (87.7%)	291 (84.3%)	1362 (87%)
	Female to male	411 (33.7%)	54 (15.7%)	204 (13%)
	Missing	5	1	6
Donor CMV	Negative	569 (46.8%)	188 (54.8%)	757 (48.6%)
	Positive	647 (53.2%)	155 (45.2%)	802 (51.4%)
	Missing	10	3	13
Donor/patient CMV	Neg/neg	291 (24.1%)	94 (27.6%)	385 (24.9%)
	Pos/neg	118 (9.8%)	30 (8.8%)	148 (9.6%)
	Neg/pos	274 (22.7%)	92 (27.1%)	366 (23.7%)
	Pos/pos	523 (43.4%)	124 (36.5%)	647 (41.8%)
	Missing	20	6	26
Cell source	BM	173 (14.1%)	32 (9.2%)	205 (13%)
	PB	1053 (85.9%)	314 (90.8%)	1367 (87%)
Conditioning	MAC	647 (52.8%)	143 (41.3)	790 (50.3%)
	RIC	579 (47.2%)	203 (58.7%)	782 (49.7%)
*In vivo* T‐cell depletion	No	471 (38.7%)	132 (38.4%)	603 (38.6%)
	Yes	747 (61.3%)	212 (61.6%)	959 (61.4%)
	Missing	8	2	10
Posttransplant cyclophosphamide	No	1055 (87.3%)	287 (83.4%)	1342 (86.4%)
	Yes	154 (12.7%)	57 (16.6%)	211 (13.6%)
	Missing	17	2	19

BM, bone marrow; CB, cord Blood; CMV, cytomegalovirus; CR, complete remission; FLT3‐wt, fms‐related tyrosine kinase 3‐wild type; FLT3‐ITD, fms‐related tyrosine kinase 3‐internal tandem duplication; MAC, myeloablative conditioning; MRD, minimum/measurable residual disease; MSD, matched sibling donor; PB, peripheral blood; RIC, reduced intensity conditioning; UD, unrelated donor.

Conditioning was MAC in 50.3% of the total study group of patients. Posttransplant cyclophosphamide was used in 13.6%, whereas 61.4% of the patients had in vivo T‐cell depletion. The majority (87%) of patients received peripheral blood stem cells. More than half (57.4%) of the patients were allografted from an unrelated donor, whereas allografts from matched sibling donors and haploidentical donors accounted for the remaining 30.8% and 11.8%, respectively. Most patients had compatible donor/recipient CMV serostatus, whereas 23.7% of the patients were CMV seropositive who had received seronegative allografts. Female to male allografts accounted for 13% of transplants. At the time of transplant, most of the patients (60.8%) were MRD‐negative. Data regarding the means of MRD assessment (Table S1) and corresponding cutoffs (Table S2) were available from 65 centers accounting for 540 patients of whom 224 were MRD‐positive: fluorescent‐activated cell sorting (FACS) (*N* = 14, 2.6%), polymerase chain reaction (PCR) (*N* = 118, 21.9%), a combination of PCR with either FACS (*N* = 185, 34.3%) or next‐generation sequencing (NGS) (*N* = 27, 5%), and a combination of all three methods (*N* = 196, 36.3%) were used to measure MRD. The MRD cutoff ranged from 10^−2^ to 10^−6^ depending on the assay used whereby 10^−2^ was achievable only with NGS which none of the centers solely depended on.

### 3.2 Posttransplant outcomes

The Day +180 cumulative incidence of acute GVHD grades II–IV and III–IV was 25.4% and 8.2%, respectively. The 2‐year cumulative incidence of chronic GVHD and extensive chronic GVHD was 35.4% and 13.8%, respectively. The 2‐year RI, NRM, LFS, OS, and GRFS were 26.3%, 14.9%, 59%, 67.7%, and 47.2%, respectively (Table 2). The leading causes of death (Table S3) were primary disease (41.6%), GVHD (21.5%), and infection (20.1%).

**TABLE 2:** Total population posttransplantation outcomes.

Outcome	Total population incidence (%)	95% Confidence interval
180+day acute GVHD		
Grade II–IV	25.4	23.2–27.6
Grade III–IV	8.2	6.9–9.7
2‐year		
Chronic GVHD	35.4	32.7–38.1
Extensive chronic GVHD	13.8	11.9–15.8
RI	26.3	23.9–28.8
NRM	14.9	13–16.9
LFS	59.0	56.2–61.7
OS	67.7	64.9–70.2
GRFS	47.2	44.4–50

GRFS, GVHD‐free, relapse‐free survival; GVHD, graft‐versus‐host disease; LFS, leukemia‐free survival; NRM, non‐relapse mortality; OS, overall survival; RI, relapse incidence.

**FIGURE 1:**
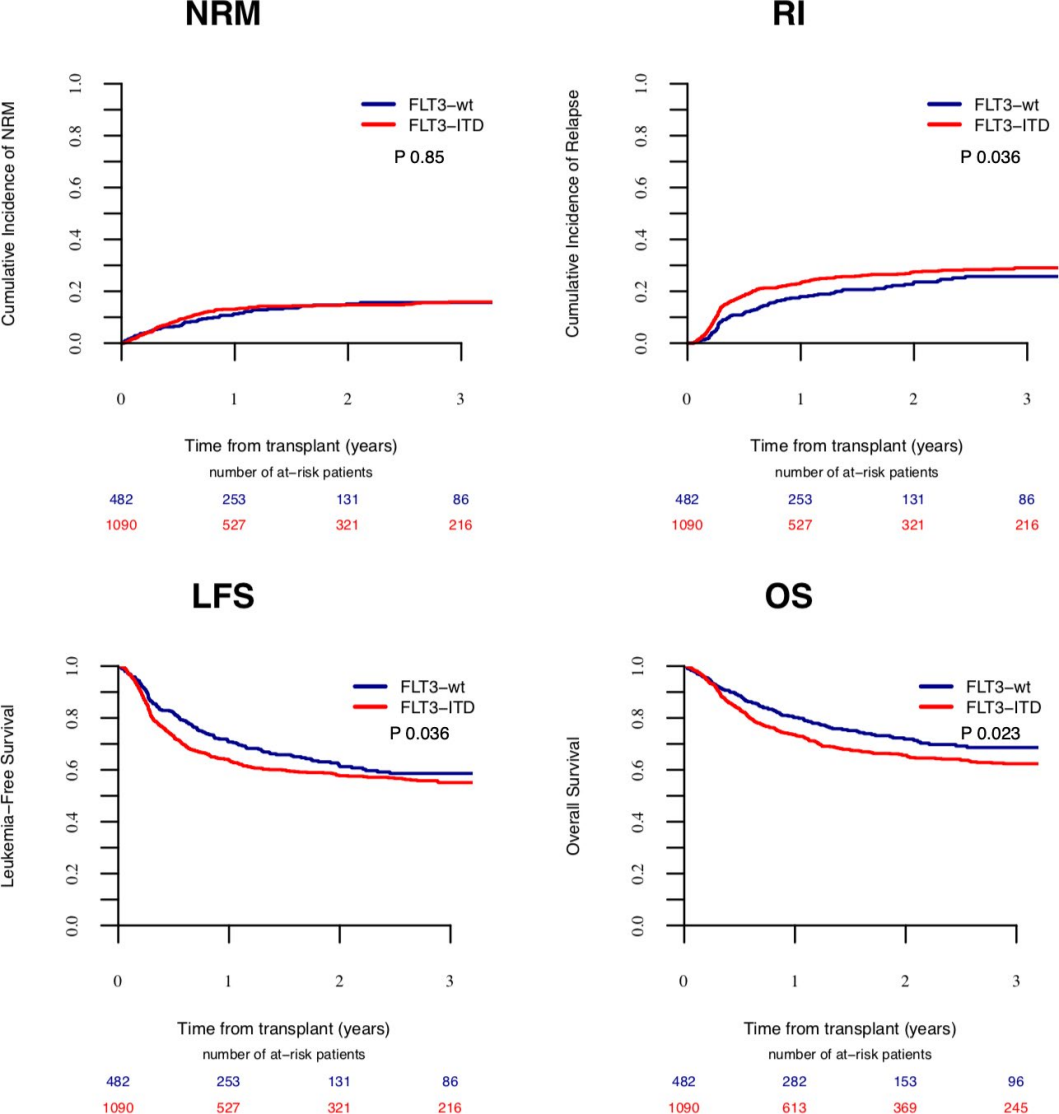
The impact of FLT3‐ITD on transplant outcome.

**FIGURE 2:**
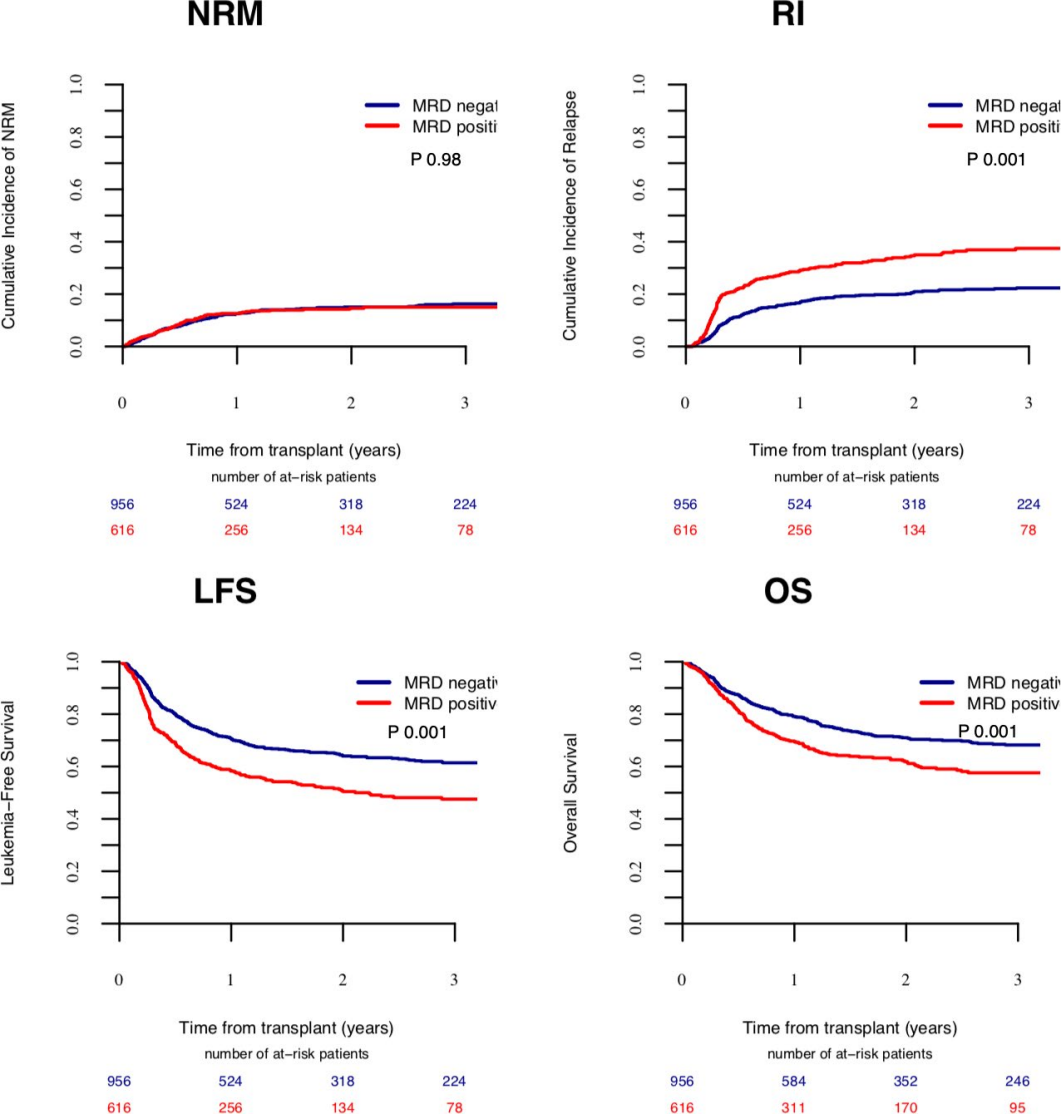
The impact of MRD on transplant outcome.

Patients in CR1 had significantly better LFS (60.6% vs. 52.5%, *p* = 0.044). The concomitant presence of *FLT3*‐ITD (Figure 1) was associated with significantly worse RI (27.5% vs. 23.6%, *p* = 0.036), LFS (57.7% vs. 61.3%, *p* = 0.036), and OS (65.6% vs. 71.9%, *p* = 0.023). Similarly, pretransplant MRD positivity (Figure 2) was associated with worse outcome in terms of RI (35% vs. 21%, *p* = 0.001), LFS (50.4% vs. 64%, *p* = 0.001), OS (61.9% vs. 71%, *p* = 0.001), and GRFS (Table S4) (39.8% vs. 51.8%, *p* = 0.001). The complete set of results of the univariate analysis are included in Tables S4 and S5.

In the multivariate analysis (Tables 3, 4 & 3, 4), patient age, *FLT3*‐ITD mutation, disease status, MRD status, KPS at transplant, donor type, female to male donor, source of stem cells, and in vivo T‐cell depletion were significant predictors of at least one transplant outcome. Increasing age was a significant negative predictor of NRM (HR 1.41, *p* < 10^−5^) and OS (HR 1.22, *p* = 0.00007). Transplantation in CR2 resulted in worse RI (HR 1.36 *p* = 0.026) and LFS (HR 1.26, *p* = 0.033), and *FLT3*‐ITD mutation negatively affected RI (HR 1.66, *p* = 0.0001), LFS (HR 1.53, *p* = 0.00006), OS (HR 1.6, *p* = 0.0001), GFRS (HR 1.34, *p* = 0.002), and incidence of acute GVHD grades II–IV (HR 1.3, *p* = 0.044). Similarly, MRD positivity at the time of transplant negatively affected RI (HR 2.18, *p* < 10^−5^), LFS (HR 1.71, *p* <10^−5^), OS (HR 1.61, *p* < 10^−5^), and GRFS (HR 1.57, *p* < 10^−5^). Conversely, a KPS score ≥90 positively impacted RI (HR 0.74, *p* = 0.012), NRM (HR 0.65, *p* = 0.006), LFS (HR 0.7, *p* = 0.0002), OS (HR 0.73, *p* = 0.004), and GRFS (HR 0.76, *p* = 0.001). Allografts from unrelated and haploidentical donors adversely affected NRM (HR 1.79, *p* = 0.03, and HR 2.63, *p* = 0.0001, respectively), and OS (HR 1.35, *p* = 0.012 and HR 1.45, *p* = 0.037, respectively). In addition, an allograft from an unrelated donor increased the incidence of acute GVHD grades II–IV (HR 1.35, *p* = 0.018), whereas one from a haploidentical donor decreased extensive chronic GVHD (HR 0.53, *p* = 0.03). A female to male donor resulted in significantly increased acute GVHD grades II–IV (HR 1.41, *p* = 0.015) and peripheral blood stem cells negatively impacted the incidence of chronic GVHD (HR 1.69, *p* = 0.001) and extensive chronic GVHD (HR 2.57, *p* = 0.0007) and as such, worsened GRFS (HR 1.42, *p* = 0.004). In vivo T‐cell depletion significantly decreased the incidence of acute GVHD grades II–IV and III–IV (HR 0.75, *p* = 0.015 and HR 0.64, *p* = 0.032, respectively), and the incidence of chronic GVHD and extensive chronic GVHD (HR 0.61, *p* = 0.00001 and HR 0.4, *p* < 10^−5^, respectively), and improved GRFS (HR 0.79, *p* = 0.006).

**TABLE 3:** Multivariate analysis of posttransplant GVHD outcome.

	Acute GVHD II–IV		Acute GVHD III–IV		Chronic GVHD		Ext. chronic GVHD	
	HR (95% CI)	*p* value	HR (95% CI)	*p* value	HR (95% CI)	*p* value	HR (95% CI)	*p* value
CR2 versus CR1	1.04 (0.79–1.36)	0.78	1.2 (0.76–1.89)	0.42	1.23 (0.95–1.58)	0.11	0.87 (0.58–1.3)	0.49
*FLT3*‐ITD	1.3 (1.01–1.67)	0.044	1.37 (0.88–2.13)	0.16	0.99 (0.79–1.24)	0.92	0.81 (0.57–1.14)	0.23
MRD positivity at transplant	1.14 (0.92–1.4)	0.24	1.26 (0.87–1.82)	0.22	1.08 (0.87–1.33)	0.49	1.3 (0.95–1.78)	0.11
Age (per 10 years)	0.96 (0.87–1.05)	0.37	0.94 (0.8–1.12)	0.51	1.01 (0.92–1.11)	0.79	1.04 (0.9–1.21)	0.60
Female to male allograft	1.41 (1.07–1.87)	0.015	1.35 (0.83–2.2)	0.23	1.04 (0.78–1.38)	0.79	1.13 (0.75–1.7)	0.57
MSD (reference)	1		1		1		1	
UD	1.35 (1.05–1.74)	0.018	1.46 (0.93–2.3)	0.10	0.89 (0.71–1.11)	0.31	0.86 (0.61–1.2)	0.36
Haplo	1.15 (0.81–1.65)	0.44	1.57 (0.87–2.84)	0.13	0.75 (0.53–1.07)	0.11	0.53 (0.3–0.94)	0.03
KPS≥90	1.07 (0.83–1.38)	0.60	0.7 (0.47–1.05)	0.083	0.9 (0.71–1.15)	0.40	0.86 (0.6–1.25)	0.43
PB vs BM	0.99 (0.73–1.33)	0.92	1.74 (0.92–3.29)	0.087	1.69 (1.22–2.33)	0.001	2.57 (1.49–4.44)	0.0007
RIC vs MAC	0.91 (0.72–1.15)	0.44	1.16 (0.77–1.75)	0.48	0.96 (0.77–1.21)	0.75	1.12 (0.79–1.6)	0.53
*in vivo* TCD	0.75 (0.59–0.95)	0.015	0.64 (0.42–0.96)	0.032	0.61 (0.49–0.76)	0.00001	0.4 (0.29–0.56)	<10–5

BM, bone marrow; CR, complete remission; GVHD, graft‐versus‐host disease; GRFS, GVHD‐free, relapse‐free survival; Haplo, haploidentical donor; KPS, Karnofsky performance score; MAC, myeloablative conditioning; MRD, minimal/measurable residual disease; MSD, matched sibling donor; PB, peripheral blood; RIC, reduced intensity conditioning; TCD, T‐cell depletion; UD, unrelated donor.

**TABLE 4:** Multivariate analysis of posttransplant outcome.

	RELAPSE		NRM		LFS		OS		GRFS	
	HR (95% CI)	*p* value	HR (95% CI)	*p* value	HR (95% CI)	*p* value	HR (95% CI)	I value	HR (95% CI)	*p* value
CR2 versus CR1	1.36 (1.04–1.77)	0.026	1.08 (0.76–1.55)	0.66	1.26 (1.02–1.57)	0.033	1.17 (0.92–1.49)	0.20	1.19 (0.98–1.44)	0.08
*FLT3*‐ITD	1.66 (1.28–2.16)	0.0001	1.28 (0.91–1.81)	0.15	1.53 (1.24–1.88)	0.00006	1.6 (1.26–2.03)	0.0001	1.34 (1.12–1.6)	0.002
MRD positivity at transplant	2.18 (1.76–2.71)	<10–5	1.07 (0.8–1.45)	0.64	1.71 (1.44–2.04)	<10–5	1.61 (1.32–1.96)	<10–5	1.57 (1.34–1.82)	<10–5
Age (per 10 years)	0.93 (0.84–1.02)	0.14	1.41 (1.21–1.64)	<10–5	1.06 (0.98–1.15)	0.14	1.22 (1.1–1.34)	0.00007	1.03 (0.96–1.11)	0.40
Female to male allograft	0.86 (0.62–1.19)	0.36	1.26 (0.85–1.87)	0.25	0.99 (0.77–1.27)	0.93	1.07 (0.81–1.42)	0.62	1.08 (0.87–1.34)	0.47
MSD (reference)	1		1		1		1		1	
UD	0.91 (0.71–1.16)	0.44	1.79 (1.22–2.63)	0.003	1.12 (0.92–1.38)	0.26	1.35 (1.07–1.71)	0.012	1.06 (0.89–1.26)	0.51
Haplo	0.7 (0.47–1.06)	0.089	2.63 (1.61–4.3)	0.0001	1.14 (0.84–1.54)	0.41	1.45 (1.02–2.05)	0.037	0.9 (0.69–1.18)	0.45
KPS≥90	0.74 (0.58–0.94)	0.012	0.65 (0.48–0.88)	0.006	0.7 (0.58–0.85)	0.0002	0.73 (0.59–0.91)	0.004	0.76 (0.64–0.9)	0.001
PB vs BM	1.21 (0.87–1.7)	0.26	1.05 (0.68–1.63)	0.81	1.15 (0.88–1.5)	0.29	1.11 (0.82–1.49)	0.51	1.42 (1.12–1.81)	0.004
RIC vs MAC	1.16 (0.91–1.48)	0.24	1.03 (0.75–1.43)	0.84	1.11 (0.91–1.34)	0.30	1.16 (0.93–1.44)	0.18	1.1 (0.93–1.31)	0.26
*in vivo* TCD	0.99 (0.77–1.26)	0.92	1.13 (0.8–1.6)	0.48	1.03 (0.84–1.26)	0.76	1.07 (0.85–1.34)	0.56	0.79 (0.67–0.94)	0.006

BM, bone marrow; CR, complete remission; GRFS, GVHD‐free, relapse‐free survival; Haplo, haploidentical donor; KPS, Karnofsky performance score; LSF, leukemia‐free survival; MAC, myeloablative conditioning; MRD, minimal/measurable residual disease; MSD, matched sibling donor; NRM, non‐relapse mortality; OS, overall survival; PB, peripheral blood; RIC, reduced intensity conditioning; TCD, T‐cell depletion; UD, unrelated donor.

In the subgroup univariate analysis by disease status at transplant (Table S6), a concomitant *FLT3*‐ITD mutation in patients in CR1 had a similar negative effect on RI (26.8% vs. 19%, *p* = 0.01), LFS (59.1% vs. 66.1%, *p* = 0.019), and OS (66.6% vs. 75.2%, *p* = 0.032). Similarly, MRD positivity had a similar negative effect in this subgroup on RI (34% vs. 20.1%, *p* = 0.001), LFS (52.7% vs. 65.1%, *p* = 0.001), OS (61.6% vs. 72.2%, *p* = 0.001), and GRFS (42.1% vs. 52.9%, *p* = 0.001). In patients in CR2, *FLT3*‐ITD was also associated with worse LFS (46.4% vs. 55.6%, *p* = 0.025), OS (57.7% vs. 67.6%, *p* = 0.018), and GRFS (35.4% vs. 43.5%, *p* = 0.023) and MRD positivity was associated with higher RI (37.9% vs. 24.6%, *p* = 0.037) and lower LFS (43.5% vs. 59.4%, *p* = 0.014). When stratified according to *FLT3*‐ITD mutation status (Table S7), patients harboring the mutation with MRD positivity at transplant had higher acute II–IV (32.8% vs. 24.8%, *p* = 0.005) and III–IV (11.8% vs. 7.4%, *p* = 0.02) GVHD and RI (38.7% vs. 22%, *p* = 0.001) with lower LFS (46.9% vs. 63%, *p* = 0.001), OS (55.8% vs. 70.3%, *p* = 0.001), and GRFS (36.7% vs. 51.7%, *p* = 0.001). MRD positivity had a similar effect in patients without *FLT3*‐ITD mutation and was associated with worse RI (29.6% vs. 17.7%, *p* = 0.001) and LFS (55.3% vs. 67.1%, *p* = 0.003).

## 4 DISCUSSION

*NPM1*‐mutated AML accounts for more than 50% of normal karyotype AML and while the individual impact of MRD, disease status, and *FLT3*‐ITD has been investigated, little is known about their combined respective contributions in influencing posttransplant outcome.^5^, ^7^ In this retrospective EBMT registry‐based study, we evaluated the predictive factors of posttransplant outcomes and the individual and aggregate impact of *FLT3*‐ITD mutation, disease status, and MRD at transplant at transplant in 1572 adult (age ≥18 years) patients known to have *NPM1*‐mutated normal karyotype AML. We found that a concomitant *FLT3*‐ITD mutation, MRD positivity before transplant, transplantation in CR2, and KPS <90 increased RI and thus significantly impacted LFS and OS (Table 4). Our analysis highlighted the negative impact each of the three factors had independently of each other and without significant inter‐factor interactions.

Since its discovery in 2005, *NPM1* mutation in normal karyotype AML has been shown to infer a positive prognostic effect and a major survival advantage in the absence of *FLT3*‐ITD.^8^, ^19^ In allografted patients, one EBMT registry study showed that concomitant *FLT3*‐ITD resulted in significant decrease in LFS from 81% to 66%.^11^ Here, we confirmed the negative impact of *FLT3*‐ITD on RI (HR 1.66, *p* = 0.0001), LFS (HR 1.53, *p* = 0.00006), OS (HR 1.6, *p* = 0.0001), and GRFS (HR 1.34, *p* = 0.002) (Table 4). The *FLT3*‐ITD fragment lengths and allelic ratios were not uniformly available for all patients in the registry, thus limiting their incorporation in our analysis. Given the central role allelic ratios play in the 2017 ELN risk stratification recommendations, we can speculate that the impact of *FLT3*‐ITD is even larger had we been able to identify patients with high allelic ratio.^9^

MRD before transplant had a significant impact on RI (HR 2.18, *p* < 10^−5^), LFS (HR 1.71, *p* < 10^−5^), OS (HR 1.61, *p* < 10^−5^), and GRFS (HR 1.57, *p* < 10^−5^) (Table 4). The significant association of MRD with outcome was irrespective of *FLT3*‐ITD and its effect on RI and LFS was independent of disease status. On the other hand, MRD did not appear to have a significant association with OS and GRFS in patients in CR2 given the relatively more advanced underlying disease in this patient population. Some studies have independently demonstrated the prognostic value of MRD in influencing RI and thus survival in AML patients.^12^, ^13^, ^20^ In lieu of such results, the ELN introduced a new category of disease, CR without MRD.^9^, ^21^, ^22^ This said, a recent EBMT survey of 106 transplant centers across 29 countries demonstrated that 92 centers have been assessing MRD routinely for AML since 2010 using different techniques such as PCR, multi‐parameter flow cytometry (MFC), PCR+MFC, PCR+NGS, or all three whereby the majority depended on a combination of MFC and PCR which is comparable to our analysis.^23^ The survey, a step toward the routine inclusion of MRD status in acute leukemia, also highlighted the frequent reassessment of MRD adopted by centers whereby the majority assessed MRD every 2–3 months for 2 years (range: 1 year––until relapse) which stresses the value of MRD posttransplant as well.^23^

Patients with normal karyotype, *NPM1*‐mutated AML without *FLT3*‐ITD, who belong to the ELN favorable risk group, are generally referred to allo‐HCT in CR2 or beyond. In our study, transplantation in CR2, as opposed to CR1, resulted in a significant negative impact on RI (HR 1.36, *p* = 0.026) and thus, LFS (HR 1.26, *p* = 0.033) irrespective of other predictive factors including MRD status and *FLT3*‐ITD mutational status. These results are in agreement with a previous retrospective study that analyzed 156 patients with *NPM1*‐mutated AML without *FLT3*‐ITD, which demonstrated that both CR2 and advanced disease at transplant, negatively affected RI (HR 3.65 *p* = 0.02 and HR 5.73 *p* = 0.0002, respectively), LFS (HR 2.53 *p* = 0.005 and HR 3.94 *p* = 0.0002, respectively), and OS (HR 2.30 *p* = 0.02 and HR 3.90 *p* = 0.01, respectively).^10^ This of course, does not imply that all patients harboring the mutation ought to be transplanted in CR1 as the majority of patients with favorable cytogenetics are likely to achieve cure without HCT. Instead, it highlights the rather worse survival outcome expected in patients who end up requiring HCT in CR2.

On the other hand, the indication for allo‐HCT in CR1 in AML patients with *NPM1* mutation *and FLT3*‐ITD low allelic ratio, who also belong to the ELN favorable risk group remains controversial.^9^, ^24^ Many centers refer to transplant only patients with persistent MRD positivity. Yet, our study highlights the persistent negative predictive value of MRD positivity even in the transplant setting. These patients may benefit from prophylactic use of *FLT3* tyrosine kinase inhibitors (TKIs).^25^, ^26^, ^27^, ^28^, ^29^, ^30^, ^31^

There is currently, very little is known about the respective contributions of disease status, MRD status, and *FLT3*‐ITD to posttransplantation outcome in *NPM1*‐mutated AML. Given the grave prognosis on OS that relapse imposes in this patient population and the relatively high incidence of relapse, it is rather important to emphasize factors predictive of relapse to identify high‐risk patients who could benefit from personalized treatment approaches such as prophylactic use of *FLT3* TKIs or more frequent follow‐up.

## 5 LIMITATIONS

The limitations of this analysis include the retrospective study design, the absence of a unified MRD measurement technique among centers, and the unavailability of molecular MRD assessment, specifically *NPM1* or *FLT3* MRD. The use of different assays to assess MRD resulted in a wide range of MRD cutoffs utilized by different centers. Furthermore, MRD testing techniques were available for centers as a whole, but data about the techniques used and their results for individual patients were not available, therefore limiting further analysis. The limited MRD technique data, although limiting, are important for solidifying the fact that patients were assessed for MRD according to universally accepted definitions, with the majority of the patients assessed by two modalities. In addition, it would have been valuable to analyze molecular *NPM1* MRD which has been shown to be a significant predictor of relapse despite the favorable prognostication that the *NPM1* mutation infers.^20^ This could help highlight patients who could potentially benefit from additional chemotherapy. Posttransplant MRD was also not available in the registry, which has been shown, in combination with pretransplant MRD as a better predictor of outcome compared to pretransplant MRD alone.^32^ Furthermore, additional variables such as pretreatment patient characteristics including comorbidities, *FLT3*‐ITD fragment length, and *FLT3*‐ITD allelic ratio were also unavailable for investigation, further limiting the analysis. This is important since the *FLT3*‐ITD allelic ratio is vital in allocating ELN risk and aiding treatment decision. Finally, whether patients harboring *FLT3*‐ITD actually received treatment with *FLT3*‐inhibitors or whether venetoclax combinations were utilized given their possible promising role in *NPM1*‐mutated disease was also unavailable.^33^

## 6 CONCLUSION

We demonstrate that in adult patients with normal karyotype *NPM1*‐mutated AML, *FLT3*‐ITD mutation status, disease status at transplant, MRD status at transplant, and KPS significantly and independently influence RI and LFS and thus OS. Knowledge of factors that substantially influence posttransplant outcomes allows physicians to undertake, when feasible, an alternative treatment approach to decrease the risk of relapse and thus improve the survival.

## CONFLICT OF INTEREST

The authors declare no potential conflict of interest.

## DISCLOSURES

Dr. Jindra Pavel was supported by *the Ministry of Health of Czech Republic under Grant AZV NV18*‐*03*–*00277*.

## AUTHOR CONTRIBUTIONS

R.A.: data curation, investigation, project administration, writing original draft, and editing/reviewing; M.L.: data curation, formal analysis, methodology, and review; E.D., R.N., A.H., G.S., M.S., J.H.B., N.K., E.T., G.C., X.P., H.M., MT.R., P.J., D.B., D.B., H. LW., and A.N: data acquisition, investigation, validation, and review; A.B. and M.M.: conceptualization, investigation, supervision, validation, and review.

## Supporting information

Table S1

Table S2

Table S3

Table S4

Table S5

Table S6

Table S7

Appendix

## Data Availability

Data were provided and approved by the Acute Leukemia Working Party (ALWP) of the EBMT. The EBMT is a voluntary collaborative working group that includes more than 600 transplant centers that report all consecutive stem cell transplantations and follow‐up once a year, with regular audits to determine and maintain the accuracy of the data.
